# An Energy Efficient Technique Using Electric Active Shielding for Capacitive Coupling Intra-Body Communication

**DOI:** 10.3390/s17092056

**Published:** 2017-09-08

**Authors:** Chao Ma, Zhonghua Huang, Zhiqi Wang, Linxuan Zhou, Yinlin Li

**Affiliations:** 1 School of Mechatronical Engineering, Beijing Institute of Technology, Beijing 100081, China; 20081124@bit.edu.cn (C.M.); huangzh@bit.edu.cn (Z.H.); wangzhiqi@bit.edu.cn (Z.W.); 2 The Bradley Department of Electrical and Computer Engineering, Virginia Polytechnic Institute and State University, Blacksburg, VA 24061, USA; charlesz@vt.edu

**Keywords:** intra-body sensor networks, capacitive coupling, electric active shielding, energy efficiency, finite element method (FEM)

## Abstract

Capacitive coupling intra-body communication (CC-IBC) has become one of the candidates for healthcare sensor networks due to its positive prevailing features of energy efficiency, transmission rate and security. Under the CC-IBC scheme, some of the electric field emitted from signal (SIG) electrode of the transmitter will couple directly to the ground (GND) electrode, acting equivalently as an internal impedance of the signal source and inducing considerable energy losses. However, none of the previous works have fully studied the problem. In this paper, the underlying theory of such energy loss is investigated and quantitatively evaluated using conventional parameters. Accordingly, a method of electric active shielding is proposed to reduce the displacement current across the SIG-GND electrodes, leading to less power loss. In addition, the variation of such loss in regard to frequency range and positions on human body was also considered. The theory was validated by finite element method simulation and experimental measurement. The prototype result shows that the receiving power has been improved by approximate 5.5 dBm while the total power consumption is maximally 9 mW less using the proposed technique, providing an energy efficient option in physical layer for wearable and implantable healthcare sensor networks.

## 1. Introduction

Intra-body Communication (IBC) provides an appealing wireless connection of wearable and implantable biomedical sensors for ubiquitous monitoring of the patient’s physiological parameters. Due to its energy efficiency and security features [1,2], IBC was selected as a physical layer candidate in the IEEE 802.15.6 (WBAN) standard. IBC, which uses body tissue as the signal transmission medium, is generally divided into two categories: galvanic coupling and capacitive coupling [3]. Galvanic coupling uses electrodes in direct contact with the skin of the subject [4,5], whereas the signal electrode of the capacitive coupling can be either attached to the skin directly or separated by clothes or insulators from the skin [6]. In addition, capacitive coupling has a higher gain and a relatively higher frequency range of operation, between 1 and 100 MHz, which enables higher data transmission rates [7,8,9,10] and less power consumption [1] than the galvanic coupling method. As a result, capacitive coupling has been one of the most active research topics in the IBC field, mainly focusing on channel modeling [10,11,12,13,14,15,16], measurement [17,18], physical principle analysis and prototype development [19,20] to date.

**Figure 1:**
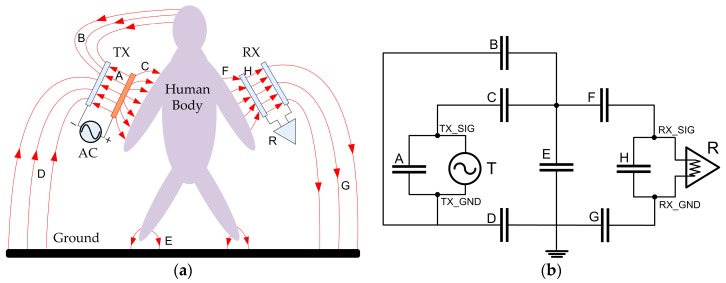
(**a**) The conventional electric field model; (**b**) The circuit model of capacitive coupling IBC.

The conventional capacitive coupling IBC, depicted in Figure 1, was put forwarded by Zimmerman in 1995 [21]. For the electric field model in Figure 1a, the signal (SIG) electrodes of transmitter and receiver are attached to the body while the ground (GND) electrodes are left floating in the air. In this case, human body is modeled as the node of a perfect conductor, and the electric couplings among the electrodes of the transceiver, body and ground plane are modeled as capacitors. In Figure 1b, an equivalent circuit model is presented accordingly. As shown in the figure, the capacitances of the various electrode pairs play a critical role to determine the path transmission gain, which significantly affects both size and power consumption of the IBC system. Therefore, the configuration of the electrodes is clearly considered to be the most important consideration in the IBC systems [1] .

Several studies have been conducted on the electrode configuration of capacitive coupling IBC. In [13], the impedance of capacitive IBC transmitter’s electrodes for both signal-ground and electrode–skin was provided. The transmission gains of the capacitive IBC channel were investigated in [15] with the frequency range from 100 kHz to 100 MHz, respecting to horizontal and vertical electrode setups as well as three electrode material. The energy loss due to the return path parasitic capacitance between the transmitter/receiver’s GND electrodes and the external ground plane was studied in [22]. However, there has been a lack of research on the effect of the parasitic capacitance between SIG and GND electrodes of the transmitter, designated as equivalent capacitor *A* in Figure 1. The parasitic capacitor *A*, connected parallel to the signal source, could potentially leak the displacement current directly from the SIG to GND electrode, which can lead to an internal power consumption increase and a transmission gain loss. While the parasitic capacitance between the SIG-GND electrodes of the transmitter has been identified explicitly in [12,13,21,23], their influences on the power consumption and transmission gain as well as corresponding solutions have not been fully studied so far.

In this study, our first aim is to analyze the power consumption caused by the parasitic capacitance between transmitter electrodes of the capacitive coupling IBC. Secondly, we intend to propose an electrode configuration using the scheme of capacitive reflector [24] to reduce such power loss. Considering the fact that the vertical electrodes (parallel-plate structure) [1,12,13,22,23,24,25] of the transmitter is the optimal configuration in regard to the transmission gain [1] and system size, as well as that the vertical electrode has minimal plates distance and hence a larger amount of energy loss in the system, therefore our study focuses on the vertical electrode structure of the transmitter.

This paper is organized as follows: Section 2 presents the effect of the parasitic capacitance and the theory of the electric active shielding from the perspective of circuit model. In Section 3, the finite-element method (FEM) model of a capacitive coupling IBC is established and simulated to evaluate the effect of the shield electrode from the angle of electric field strength distribution. Section 4 presents an experimental validation of the shield electrode using a prototype transmitter powered by battery. Section 5 presents the final conclusions of this paper.

## 2. Effect of Electrodes Parasitic Capacitance and Electric Active Shielding

**Figure 2:**
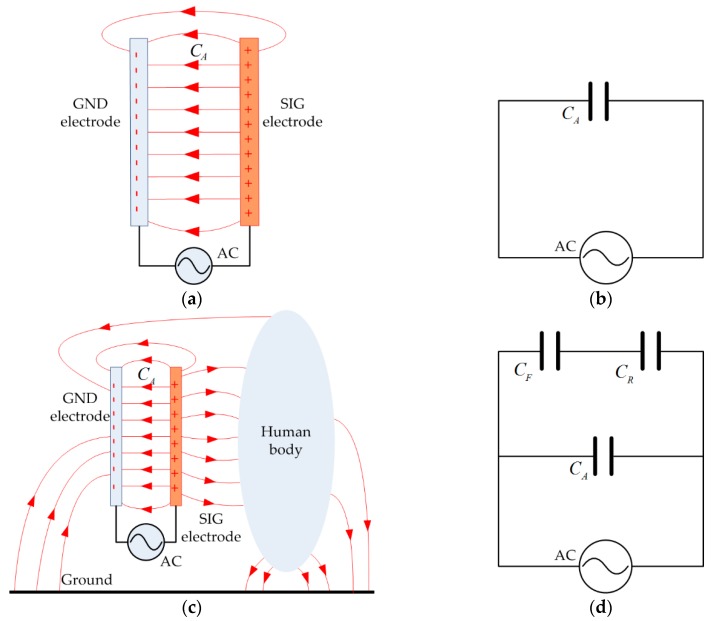
The model of the parasitic capacitances on the transmitter electrodes of IBC system. (**a**) The parasitic capacitance between SIG-GND electrodes; (**b**) Simplified circuit model of (**a**); (**c**) The parasitic capacitances among electrodes, human body and ground plan; (**d**) Simplified circuit model of (**c**).

The vertical electrode plate pair in parallel structure is conceptually equivalent to a capacitor. Ideally, the parasitic capacitance of the vertical electrode in free space is proportional to the area of its plates and inversely proportional to the plate separation. When an alternating signal source is applied to the plates, a displacement current flow through them, as illustrated in Figure 2a. The equivalent circuit model is presented in Figure 2b, where the parallel-plate is modeled as capacitor *C_A_*. When the parallel-plate is placed on human body as depicted in Figure 2c, the current supplied by the signal source is divided into two branches: some of the current flows across the parallel-plate directly, and the rest flows through all the way passing the forward path of human body and then the return path from ground plane to the negative port of the signal source. Accordingly, a simplified circuit model is built in Figure 2d, where *C_F_* and *C_R_* are the equivalent capacitors of forward path and return path, respectively. Obviously, the capacitor *C_A_* divides a portion of the current away from the branch with *C_F_*, resulting in a power loss in the same way as the internal impedance of a signal source. Therefore, it is desirable to find a solution to minimize the internal displacement current (IDC) flowing across the capacitor *C_A_* for the purpose of reducing power consumption. 

**Figure 3:**
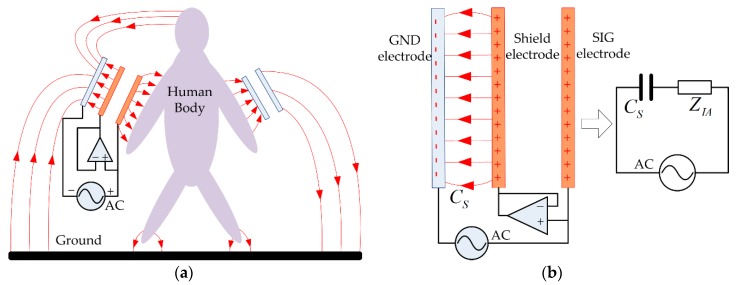
The principle of the shield electrode applied to the IBC transmitter electrodes. (**a**) The capacitive-coupled IBC with the shield electrode; (**b**) Model of the shield electrodes along with its simplified circuit.

Inspired by the concept of electric active shielding (or capacitive reflector) proposed by National Aeronautics and Space Administration’s (NASA) Goddard Space Flight Center [24], a capacitive shield electrode is introduced into the electrode configuration of the IBC transmitter in order to reduce the IDC, as depicted in Figure 3. The objective of NASA’s capacitive reflector is to develop a proximity sensing skin that would allow a robot to sense intruding objects without blind spots up to one foot. It uses a capacitive sensing element backed by a reflector element, which is driven by the same voltage as the sensing electrode. Acting as a shield to reflect all field lines away from the grounded robot arm, the range of the sensor can be extended. In our study, the shield is used for the purpose of reducing the IDC instead; therefore the power loss for the IBC system can be reduced.

As illustrated in Figure 3a, the shield electrode for IBC transmitter has the same size as the GND electrode, and it is placed between the SIG-GND electrodes. Moreover, an isolation amplifier (IA) is deployed to connect the SIG and shield electrode, keeping the voltage of the two electrodes in the same amplitude and phase. The capacitance between the GND and shield electrodes and the input impedance of IA are referred as *C_S_* and *Z_IA_*, respectively. The model of the shielded transmitter electrodes along with its simplified circuit are built in Figure 3b. As can be seen, the *C_S_* and *Z_IA_* are connected in series, and hence the current of this branch decreases when the *Z_IA_* is introduced. Since the input impedance of an IA is up to 10 GΩ, the IDC flowing from the SIG to GND electrode becomes negligible.

**Figure 4:**
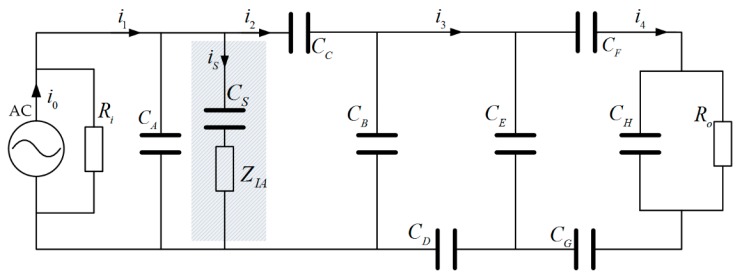
Simplified conventional IBC lumped circuit.

In order to exemplify the effect of the shield electrode quantitatively, the ratio of receiving current to the source, or the current transmission gain, is investigated based on the conventional model by Zimmerman. The circuit of Figure 1b can be further described as Figure 4, in which the capacitor *C_A_* is replaced by *C_S_* and *Z_IA_* in serial when the shield electrode is investigated. 

Since the signal source and the capacitor *C_A_* are connected in parallel, the current source can be used for analysis. According to the Kirchhoff’s current law, the relation of the currents in Figure 4 is represented as follows:

(1) $$\left\{ \begin{array}{l} i_{4}/i_{3}=Z_{E}/\left(Z_{E}+Z_{1}\right)\\ i_{3}/i_{2}=Z_{B}/\left(Z_{B}+Z_{2}\right)\\ i_{2}/i_{1}=Z_{\mathrm{int}}/\left(Z_{\mathrm{int}}+Z_{3}\right)\\ i_{1}/i_{0}=R_{i}/\left(R_{i}+Z_{A}//Z_{3}\right) \end{array}\right.$$

where *Z_B_, Z_C_, Z_D_, Z_E_, Z_F_, Z_H,_ Z_G_* are the impedance of capacitors *C_B_, C_C_, C_D_, C_E_, C_F_, C_H,_ C_G_*, respectively. *Z*_1_, *Z*_2_ and *Z*_3_ can be written as:

(2) $$\left\{ \begin{array}{l} Z_{1}=Z_{F}+Z_{G}+Z_{H}//R_{o}\\ Z_{2}=Z_{D}+Z_{E}//Z_{1}\\ Z_{3}=Z_{C}+Z_{B}//Z_{2} \end{array}\right.$$

and *Z*_int_ is the impedance of the parasitic capacitance between SIG-GND electrodes, given by:

(3) $$Z_{\mathrm{int}}=\left\{ \begin{array}{l} \frac{1}{j\omega C_{A}},\;\mathrm{without}\;\mathrm{shield}\;\mathrm{electrode}\\ Z_{IA}+\frac{1}{j\omega C_{S}},\;\mathrm{with}\;\mathrm{shield}\;\mathrm{electrode} \end{array}\right.$$

In reality, the distance between the shield electrode and the GND electrode can be regarded as the one between SIG and GND, thus

(4) $$C_{A}\approx C_{S}$$

Accordingly, based on the set of equations in (1), the transfer function of the transmission current gain can be written as:

(5) $$G=\frac{i_{4}}{i_{0}}=\frac{Z_{E}Z_{B}R_{i}}{\left(Z_{E}+Z_{1}\right)\left(Z_{B}+Z_{2}\right)\left(1+Z_{3}/Z_{\mathrm{int}}\right)\left(R_{i}+Z_{A}//Z_{3}\right)}$$

As can be seen from Equation (5), the current gain is proportional to the value of *Z*_int_, indicating that the gain is improved by introducing the shield electrode. 

**Figure 5:**
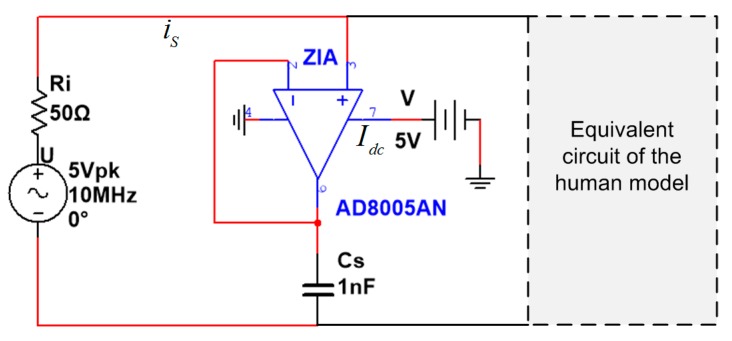
The conventional IBC lumped circuit built in Multisim software.

To evaluate the effect of the shield electrode on the power consumption, the software Multisim is used. Multisim (National Instruments, Austin, TX, USA) is an electronic schematic capture and simulation program that employs the original Berkeley SPICE software. Simulating the circuit with SPICE is the industry-standard way to verify circuit operation at the transistor level before committing to fabrication. It provides a reliable approach to achieve the power consumption of the circuit, with the consideration of the power consumed by the isolation amplifier. The conventional IBC circuit model is constructed in Multisim workbench, as illustrated in Figure 5. For analysis purpose, the signal source is transformed equivalently from current source into voltage source with an internal resistor connected in series. The chip AD8005 from Analog Device is used as the isolation amplifier, which has a bandwidth of 270 MHz and a maximal power supply consumption of 2 mW. Since the equivalent circuit of the human model and the receiver in Multisim are the same as in Figure 4, the rest of the circuit is represented as a gray rectangle in Figure 5. The total power consumption of the circuit is defined as:

(6) P={Re(UrmsIrms*),   without shield electrodeRe(UrmsIrms*)+VIdc, with shield electrode

where $U_{\mathrm{rms}}$ is the complex effective voltage of the signal source and $I^{*}{}_{\mathrm{rms}}$ is the complex conjugate of the signal source effective current, V is the DC power supply to the isolation amplifier and *I_dc_* is its supply current. 

**Figure 6:**
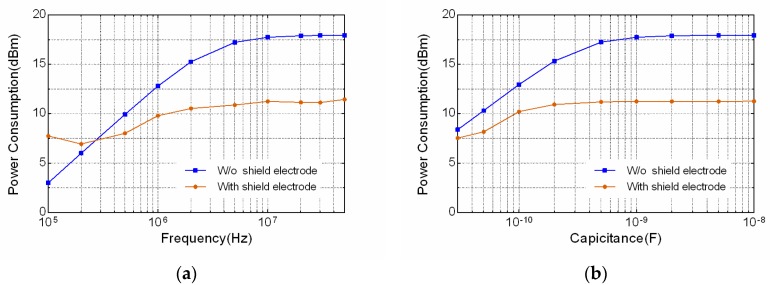
Calculated result of the total power consumption comparison between with and without shield electrode. (**a**) Power consumption with respect to frequency ranging from 100 kHz to 50 MHz; (**b**) Power consumption with respect to the capacitor *C_A_* ranging from 30 pF to 10 nF at frequency 10 MHz.

The power consumption in Equation (6) is simulated with sweeping frequencies and capacitances of transmitter electrode. As the signal frequency increases from 100 kHz to 50 MHz, the power consumption is obtained as in Figure 6a. The results show that the total power consumption is significantly reduced when frequency is larger than 300 KHz by using the shield electrode. Moreover, the capacitor *C_A_* is varied from 30 pF to 10 nF at frequency point 10 MHz, in order to emulate the various electrode configuration conditions, i.e. electrode sizes, distances, etc. The calculated result in Figure 6b exhibits that the power consumption with shield electrode is much smaller than that without the shield electrode. Therefore, the effect of parasitic capacitor *C_A_* on the power consumption is non-negligible and the shield electrode could be one of the viable solutions to reduce such losses.

It is noteworthy that, although the above result is derived based on the conventional lumped circuit model with the specific parameters of human body from Zimmerman, the conclusion can also be applicable to other distributed circuit models of human body, due to the nature of parallel connection to the signal source of the parasitic capacitance caused by electrode plates. 

## 3. Validation Using Finite Element Method

It has been proven that finite element method (FEM) can properly model the capacitive channel incorporating the electrodes of transmitter [11,12]. In order to further study the effect of shield electrode on the transmission gain in a more realistic manner, ANSYS Maxwell is employed accordingly. ANSYS Maxwell is a FEM simulation software with the capability to solve the magnetic or electric field distribution in a finite region and the volume with prescribed boundary conditions. In the study, the differences of the electric field distribution around human body between whether having the shield electrode, are compared using the Maxwell FEM software.

### 3.1. FEM Simulation Model and Setup

**Figure 7:**
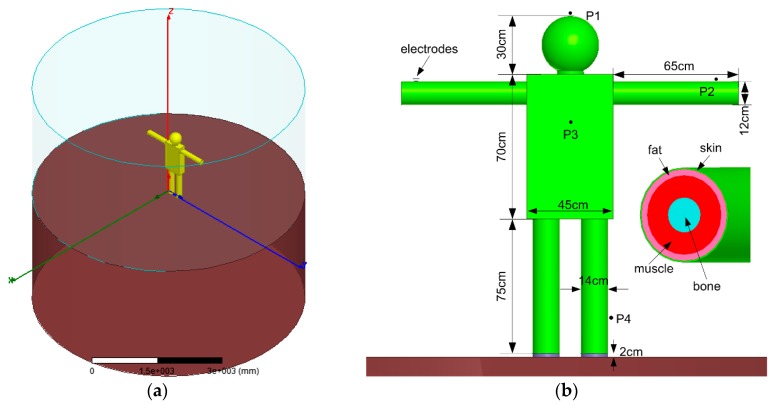
FEM model and simulation environment. (**a**) FEM model of the IBC channel with external ground. (**b**) Human body model and the transverse constituent sections.

The FEM model and simulation environment including external ground plane, air, human body and transmitter electrodes, are established in Figure 7. The ground plane is modeled as a cylinder with a diameter of 640 cm and a height of 300 cm. The human body is separated from the external ground by a 2 cm height rubber, emulating the effect of shoes. The surrounding air, also modeled as the cylinder, is designed to be the same size as the ground plane. The transmitter electrode in vertical configuration is placed on the left wrist of human body, and the signal electrode is set to be 0.5 cm above the body, in order to emulate the non-contact electrode setup. Other specifications and dimensions are denoted in Figure 7b.

The human body model is composed of head, neck, chest, arms and legs. It is modeled as a concentric cylinder or a sphere with multiple layers of skin, fat, muscles and bones, as illustrated in Figure 7b. The thicknesses of the tissue layers in different body parts are defined in Table 1, and their electrical parameters are kept the same as in [22]. 

**Figure 8:**
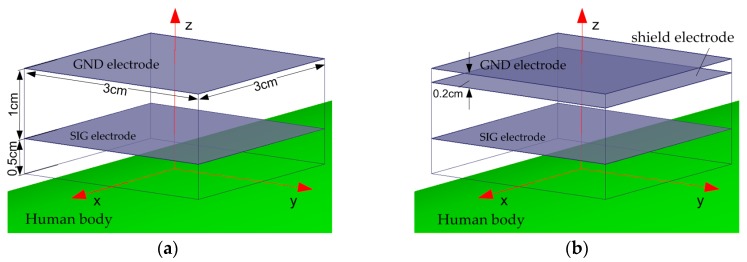
Model of transmitter electrodes in vertical configuration. (**a**) Conventional vertical electrode configuration without shield electrode. (**b**) The electrode configuration with shield electrode.

The vertical configuration of a conventional capacitive IBC electrode consists of a SIG and a GND electrode, as shown in Figure 8a. Slightly different from the conventional vertical electrode configuration, an extra electrode or shield electrode is introduced between the SIG and GND electrode, as illustrated in Figure 8b. The shield and GND electrode are in the same dimension of 3 cm × 3 cm, spaced by 0.2 cm. In order to reduce the parasitic capacitance from the edge of the SIG to GND electrode, the size of the SIG electrode is set to be smaller than the shield electrode, with a dimension of 3 cm × 3 cm. The electrodes are also made of copper with a thickness of 0.1 cm. The electrostatic solution type is selected as the solver in the 3D FEM software, and an electric potential difference of 5 $V_{rms}$ is applied as the voltage excitation.

For the configuration with shield electrode, the voltage excitation is applied to both SIG and shield electrode in order to keep the same electric potential. Moreover, in order to emulate the dispersive property of human body tissues in regard to different frequencies, the frequency-dependent dielectric constants, conductivities, and loss tangents at frequency points of 100, 200, 500 kHz, 1, 2, 5, 10, 20, 30 and 50 MHz are determined according to that in [22], and configured to be the human body tissue layers for each frequency point.

### 3.2. Simulation Results and Discussion

**Figure 9:**
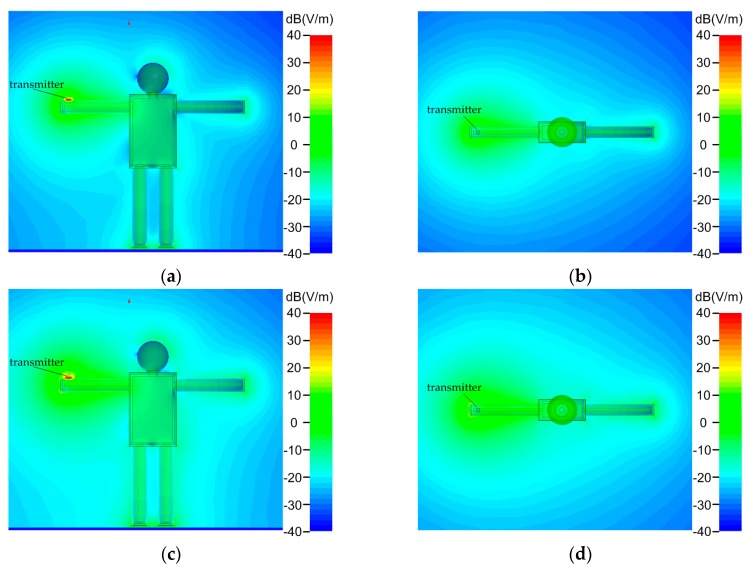
Representative electric field strength distribution in proximity to the human body with and without the shield electrode, indicating that the electric field strength is enhanced when the shield electrode is introduced. (**a**) Coronal view without shield electrode. (**b**) Transverse view without shield electrode. (**c**) Coronal view with shield electrode. (**d**) Transverse view with shield electrode.

A representative simulated result of electric field strength distribution around human body at 10 MHz is illustrated in Figure 9. 

Figure 9a shows the coronal view of the field strength distribution without the shield electrode, and Figure 9b exhibits its corresponding transverse view. Similarly, Figure 9c,d present the electric field strength distribution with the shield electrode in the coronal and transverse view respectively. By comparing the two rows, it can be found that the area with bright color around human body in Figure 9c,d tend to be bigger than the one in Figure 9a,b, implying that the electric field strength around human body is improved when the shield electrode is introduced to the transmitter.

**Figure 10:**
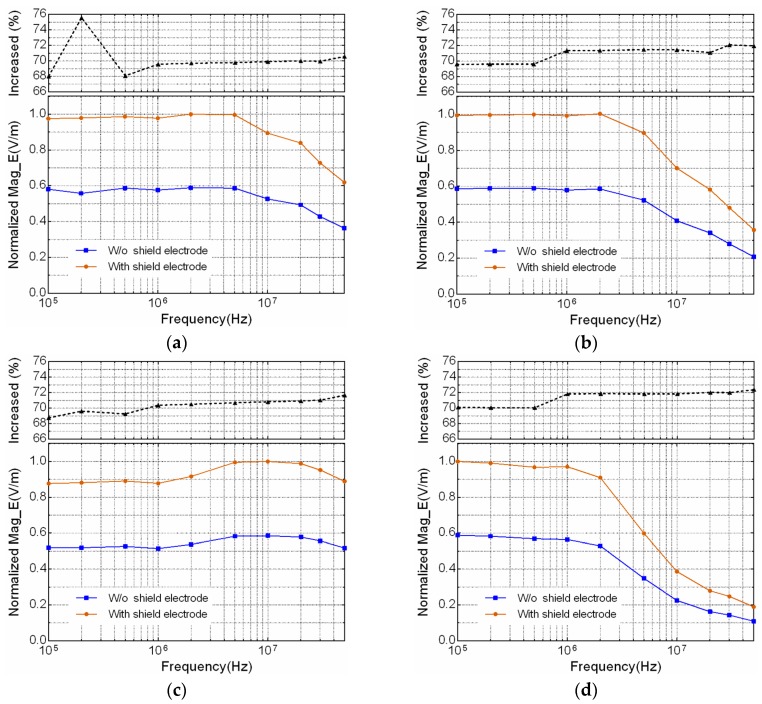
The comparison of simulated electric field strength distribution measured at 4 representative positions around the human body, with and without shield electrode. (**a**) Electric field strength distribution at position P1 (head). (**b**) Electric field strength distribution at position P2 (arm). (**c**) Electric field strength distribution at position P3 (chest). (**d**) Electric field strength distribution at position P4 (leg).

To further quantitatively investigate the effect of the shield electrode on the electric field strength distribution with respect to different frequencies, four typical positions P1 (head), P2 (arm), P3 (chest) and P4 (leg) around human body, as denoted in Figure 7b, are chosen for comparison. The simulated results are shown in Figure 10, in which the normalized electric strength values are used for comparison in condition of with and without shield electrode, and in frequency range from 100 kHz to 50 MHz. The corresponding increased percentage of electric field strength value using shield electrode is also presented on the top of each figure. Figure 10a–d show the simulated electric field strength measured at positions of P1, P2, P3 and P4, respectively. It can be seen that all four positions achieve about 70% increase of the electric field strength by using shield electrode. 

When a receiver electrode is placed on the human body, an equivalent capacitor is established between the human body and the receiver electrode. Considering the realistic context using alternating signal as the excitation source on the transmitter electrodes, a displacement current flow across the receiver electrodes. According to the rationale of the electromagnetic field, the relation of displacement current $I_{d}$ and electric field strength inside a capacitor can be written as:

(7) $$I_{d}=\varepsilon_{0}A\frac{dE}{dt}$$

where $\varepsilon_{0}$ is the permittivity, A is the area of the receiver SIG electrode and E is the electric field. As a result, the displacement current is proportional to the electric field strength. Therefore, based on the increase of the electric field strength adjacent to human body, it is concluded that the transmission gain of the IBC, in terms of displacement current, is improved by using the proposed method. 

## 4. Experimental Validation 

In order to further verify the assumption, an experiment is performed using a transmitter device with and without the shield electrode.

### 4.1. Transmitter Device

**Figure 11:**
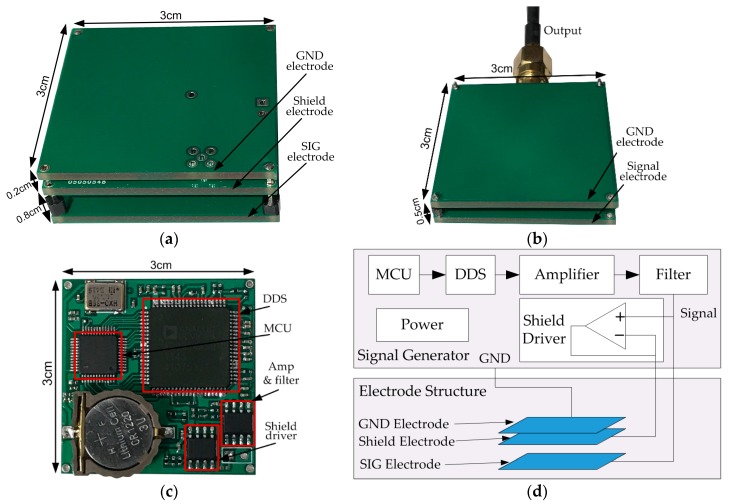
Transmitter device and receiver electrode used in the experiment. (**a**) The transmitter with the shield electrode. (**b**) The receiver electrode. (**c**) The signal source board. (**d**) Functional block diagram of the transmitter signal source board.

The transmitter is a battery-powered module composed of a 3 cm × 3 cm copper GND electrode, a 3 cm × 3 cm copper shield electrode, a 3 cm × 3 cm copper SIG electrode and an excitation signal generator board, as shown in Figure 11. When conducting the experiment without the shield electrode, the middle board in Figure 11a is removed. 

The signal generator board placed below the shield electrode is shown in Figure 11c. It consists of an AD9854 direct digital synthesizer (DDS), a STM32 micro-controller unit (MCU), an amplifying and filtering module based on AD8045, a shield driver module based on AD8045, and a button cell battery. The DDS is controlled by the MCU to provide a sine wave signal with programmable frequency ranging from 100 kHz to 50 MHz. The output sine wave is amplified and filtered, and then connected to the SIG electrode and the shielding module simultaneously. The signal voltage applied between the SIG-GND electrodes is 5 V. The detailed functional block diagram of the signal generator is shown in Figure 10d. The receiver electrode in vertical configuration, as shown in Figure 11b, is selected to pick up the signal transmitting from the source. It is made up of a SIG and a GND electrode with a size of 3 cm × 3 cm, spaced by 0.5 cm. In addition, a SMA port and a cable are used to connect to a measurement device.

### 4.2. Measurement Setup

**Figure 12:**
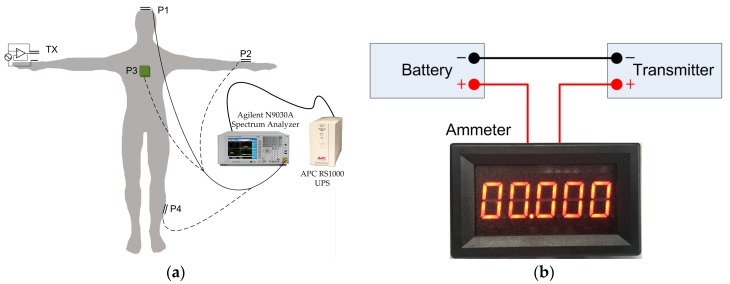
Experiment setup. (**a**) Setup to measure the received power; (**b**) Setup to measure the DC current supply of the transmitter.

The experimental equipment includes two types of battery-powered transmitters (with and without the shield electrode): the receiver, and a spectrum analyzer (Agilent N9030A, Agilent Technologies Inc, Santa Clara, CA, USA). In order to isolate the GND electrode and instrument ground, an uninterruptible Power System (UPS, APC RS1000, APC By Schneider Electric, West Kingston, RI, USA) is adopted to power up the spectrum analyzer. A male subject with height of 175 cm and weight of 75 kg is chosen for the experiment. The transmitter is placed on the wrist, and four positions (P1, P2, P3 and P4) on different parts of body are chosen to place the receiver electrode, which are then connected to the spectrum analyzer, as shown in Figure 12a. In order to evaluate the total power consumption, an ammeter (model WR5145-PR-5V, BBYE Inc., Guangzhou, China) is inserted between the positive port of battery and the transmitter, as denoted in Figure 12b. The ammeter has a current input range from 0 to 200 mA, with resolution of 0.01 mA. The power consumption is calculated by the multiplication of the measured current and battery voltage read from a multimeter. 

### 4.3. Results and Discussion

**Figure 13:**
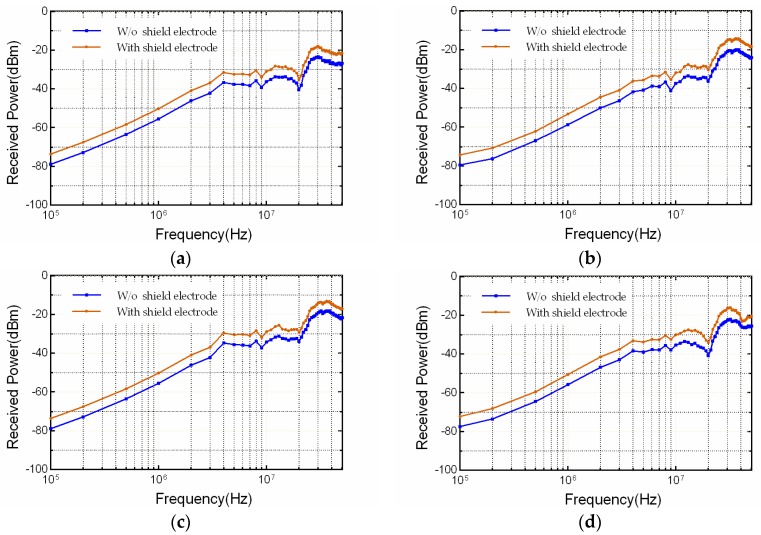
Received power comparison between the two transmitter configurations with and without the shield electrode in frequency range from 100 kHz to 50 MHz, at 4 representative positions around human body. (**a**) P1 (head), (**b**) P2 (arm), (**c**) P3 (chest), (**d**) P4 (leg).

The measured results are illustrated in Figure 13, in which Figure 13a–d present the comparison of the receiving power at four representative positions of P1 (head), P2 (arm), P3 (chest) and P4 (leg), respectively. The results are measured at the typical frequency points including 100, 200, 500 kHz, 1, 2, 5, 10, 20, 30 and 50 MHz, for both cases of with and without shield electrode on the transmitter. 

It can be found that the receiving power measured using the transmitter with shield electrode is approximately 5.5 dBm larger than the one without the shield electrode, in majority portion of the frequency range. With such observations, the measured results at the 4 positions are consistent with the simulation results, suggesting that the shield electrode between the SIG-GND electrodes of the transmitter could be an effective solution to improve the capacitive IBC transmission gain.

**Figure 14:**
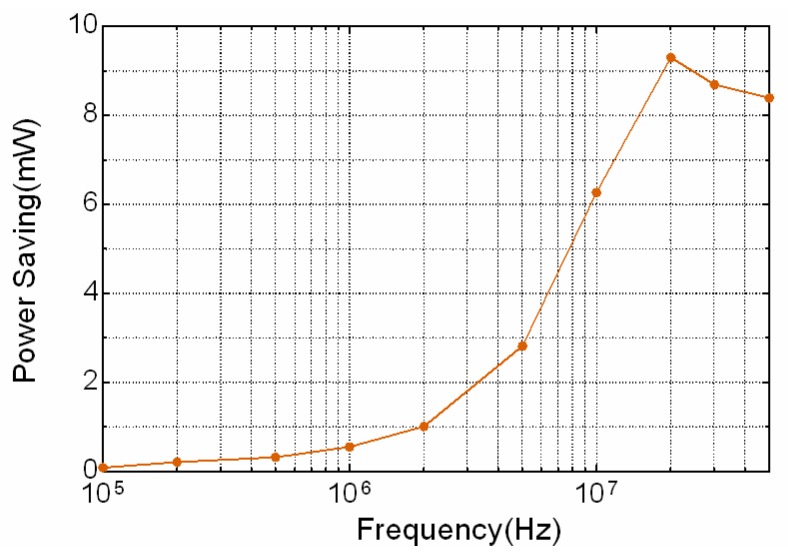
The measured average power saving in frequency range from 100 kHz to 50 MHz when using the shield electrode.

The average power saving at above representative situations is obtained by subtracting the power consumption with shield electrode from that without shield electrode. The measured power savings in frequencies ranging from 100 kHz to 50 MHz are present in Figure 14, showing that the shield electrode can reduce the total power consumption especially in higher frequency region. Meanwhile, the transmission gain is improved as well by introducing the proposed active shielding method.

## 5. Conclusions

In this paper, a method of electric active shielding for transmitter electrode is proposed, attempting to improve the power efficiency of the capacitive coupling IBC. The proposed method is firstly investigated by means of circuit analysis, using the conventional model by Zimmerman. The power consumption caused by the internal displacement current between the SIG-GND electrodes of the transmitter is evaluated and the fundamental theory of the shield electrode is explained through the circuit model. Secondly, further validation from the perspective of electric field strength distribution is conducted using ANSYS Maxwell FEM simulation software. Finally, the effect of shield electrode on the transmission gain and total power consumption is verified by experiment using battery-power transmitter together with spectrum analyzer. The simulations are implemented and compared in frequency range from 100 kHz to 50 MHz, with experiments on four representative positions around human body and in two conditions of transmitter with and without shield electrode. As a result, the simulation shows that the electric field strength in proximity to human body is improved by approximately 70% using the shield electrode. Similarly, the received power in experiment is increased by 5.5 dBm while total power consumption is reduced by 9 mW when using the shield electrode than the case without it. Both the simulation and experiment result indicate that applying shield electrode between the SIG-GND electrodes of the transmitter is a viable solution to improve the energy efficiency for capacitive coupling IBC. The proposed method can provide an energy efficient option in physical layer for wearable and implantable healthcare sensor networks.

## Figures and Tables

**Table 1:** Thicknesses of the tissue layers (mm).

	Arm	Leg	Torso	Head	Neck
Skin	1.26	1.26	1.26	1.26	1.26
Fat	8.74	8.74	8.74	2	8.74
Muscle	28	34	30	2	42
Bone	22	26	20	10	23
